# IFN-Gamma Inhibits JC Virus Replication in Glial Cells by Suppressing T-Antigen Expression

**DOI:** 10.1371/journal.pone.0129694

**Published:** 2015-06-10

**Authors:** Francesca Isabella De-Simone, Rahsan Sariyer, Yolanda-Lopez Otalora, Shadan Yarandi, Michael Craigie, Jennifer Gordon, Ilker Kudret Sariyer

**Affiliations:** Department of Neuroscience, Center for Neurovirology, Temple University School of Medicine, 3500 North Broad Street, 7th Floor, Philadelphia, PA, 19140, United States of America; University of Utah, UNITED STATES

## Abstract

**Objective:**

Patients undergoing immune modulatory therapies for the treatment of autoimmune diseases such as multiple sclerosis, and individuals with an impaired-immune system, most notably AIDS patients, are in the high risk group of developing progressive multifocal leukoencephalopathy (PML), an often lethal disease of the brain characterized by lytic infection of oligodendrocytes in the central nervous system (CNS) with JC virus (JCV). The immune system plays an important regulatory role in controlling JCV reactivation from latent sites by limiting viral gene expression and replication. However, little is known regarding the molecular mechanisms responsible for this regulation.

**Methods and Results:**

Here, we investigated the impact of soluble immune mediators secreted by activated PBMCs on viral replication and gene expression by cell culture models and molecular virology techniques. Our data revealed that viral gene expression and viral replication were suppressed by soluble immune mediators. Further studies demonstrated that soluble immune mediators secreted by activated PBMCs inhibit viral replication induced by T-antigen, the major viral regulatory protein, by suppressing its expression in glial cells. This unexpected suppression of T-antigen was mainly associated with the suppression of translational initiation. Cytokine/chemokine array studies using conditioned media from activated PBMCs revealed several candidate cytokines with possible roles in this regulation. Among them, only IFN-γ showed a robust inhibition of T-antigen expression. While potential roles for IFN-β, and to a lesser extent IFN-α have been described for JCV, IFN-γ has not been previously implicated. Further analysis of IFN-γ signaling pathway revealed a novel role of Jak1 signaling in control of viral T-antigen expression. Furthermore, IFN-γ suppressed JCV replication and viral propagation in primary human fetal glial cells, and showed a strong anti-JCV activity.

**Conclusions:**

Our results suggest a novel role for IFN-γ in the regulation of JCV gene expression via downregulation of the major viral regulatory protein, T-antigen, and provide a new avenue of research to understand molecular mechanisms for downregulation of viral reactivation that may lead to development of novel strategies for the treatment of PML.

## Introduction

Infection of glial cells by the neurotropic JC virus (JCV) causes the fatal CNS demyelinating disease, progressive multifocal leukoencephalopathy (PML), which is primarily seen in patients with underlying immunocompromised conditions [1–3]. Seroepidemiological studies have indicated that JCV infects up to 80% of human population during childhood, and establishes a latent, asymptomatic infection at multiple sites in the body, including brain, kidneys and bone marrow in healthy individuals [3–8]. Although it is considered as a rare disease, PML first received considerable attention due to an increased incidence at the onset of the AIDS pandemic. Between 3 to 5% of all HIV-infected individuals develop PML [9], [10]. Recently PML has been described in patients with autoimmune diseases treated with immunomodulatory therapies. During the last several years, PML has become a significant risk factor in multiple sclerosis patients treated with natalizumab, an anti-integrin antibody therapy [1], [11], [12]. To date, natalizumab treatment has been linked to over 500 cases of PML. PML has also been reported as a risk factor in the context of auto-immune disorders treated with a variety of other monoclonal antibody therapies, suggesting that immunosuppression may lead to reactivation of JCV in the brain and can predispose patients to the development of PML. These include rituximab (trade named Rituxan) for the treatment of B cell lymphoma and rheumatoid arthritis which targets CD20 on circulating B cells causing their depletion from periphery [13], [14] and efalizumab (trade named Raptiva) for the treatment of plaque psoriasis which targets CD11a on T cells [15].

JCV is a non-enveloped human polyomavirus with a circular double-stranded DNA genome which is composed of a bidirectional regulatory element and coding regions that produce early and late transcripts [16], [17]. The early region of JCV encodes only regulatory proteins such as T-antigen, which is required for both replication of the viral genome and transactivation of the viral promoter [17]; small t antigen (Sm t-antigen) which plays a role in viral replication cycle [18], [19]; and T’ proteins (T’135, T’136 and T’165) which are involved in viral replication [20]. The late region of JCV encodes structural capsid proteins (VP1, VP2, and VP3) and a small regulatory protein, agnoprotein. The non-coding control region of the neurotropic strains of JCV is composed of tandem repeats that have cell type-specific characteristics and activation of this type of regulatory region primarily occurs in glial cells such as oligodendrocytes and astrocytes [21]. Previous studies have demonstrated that JCV is capable of establishing a latent asymptomatic infection in the brain [6], [7], [23]. JCV DNA is easily detected in the CNS brain tissue in immunocompetent individuals demonstrating latent viral infection [22]. Interestingly, immunosuppression is associated with a significant increase in JCV DNA load in the brain [23] suggesting a possible role for neuroimmune interactions in control of viral reactivation and development of JCV-associated pathogenesis. Therefore, understanding the molecular regulation of viral reactivation leading to the development of PML is critical and further understanding of neuroimmune interactions at risk populations are needed to monitor disease progression.

Here we analyzed the possible role of immune mediators secreted by activated immune cells on JCV reactivation and replication in glial cells. Our results suggest the operation of a neuroimmune signaling between peripheral immune cells and glia mediated by IFNγ which may have a role in controlling JCV gene expression and progression of the lytic infection cycle by suppressing the major viral regulatory protein, T-antigen. These results revealed a novel signaling pathway controlling JCV reactivation in the brain, provided evidence for molecular mechanisms of JCV reactivation which may be utilized for the development of novel strategies for the treatment of PML.

## Results

### Conditioned-media from PBMCs inhibits JCV propagation in glial cells

**Fig 1:**
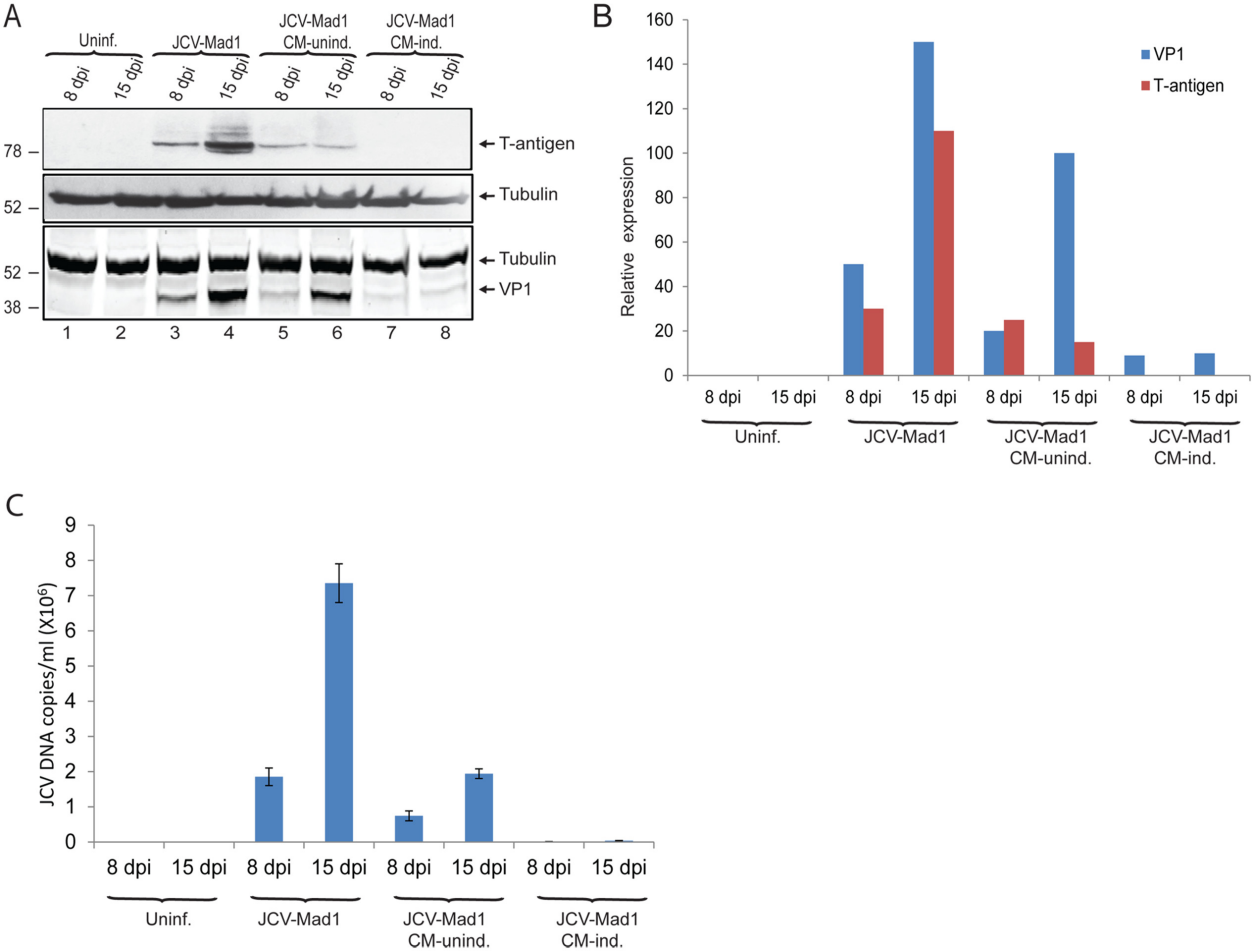
Immune mediated suppression of JC virus propagation in glial cells. A. PHFA cells were transfected/infected with the Mad1 strain of JC virus as described in Materials and Methods. At 48h post-infection, culture media of the infected cells were changed with fresh media consist of 1/1 ratio of PHFA media and conditioned media obtained from either uninduced (CM-unind.) or induced (CM-Ind.) PBMCs. The treatment was repeated at 4, 8, and 12 day post-infections. Whole cell protein lysates were prepared at 8 and 15dpi, and analyzed by Western blotting for the detection of T-antigen and VP1. B. Bar graph representation of relative T-antigen and VP1 expression normalized to tubulin from panel A. C. Q-PCR analysis of viral DNA copies in growth media of PHFA cells infected with JCV. Growth media was collected in parallel to whole cell protein extracts from the infections presented in panel B. JCV DNA copies in culture media was analyzed by Q-PCR as described in Materials and Methods.

Soluble immune mediators secreted by immune cells (cytokines and chemokines) play important roles in neuroimmune communication which provides proper signaling for host response against intracellular CNS pathogens, including viruses [24]. To investigate the possible impact of soluble immune mediators on JC virus gene expression and replication, we utilized PBMCs as the source of immune mediators in infection studies. Buffy coats were purchased from a vendor to isolate PBMCs. Serum samples were tested for viral DNA by Q-PCR analysis, and PBMCs from donors who were positive for JCV DNA were excluded from the study. PBMCs were either induced or left uninduced in culture for 48hrs as described in the Material and Methods. Conditioned media which contained soluble immune mediators secreted by PBMCs were collected and supplemented into growth media (50%) of primary human fetal astrocytic (PHFA) cells infected with JCV. Whole cell protein extracts and growth media from the cells were collected at 8 and 15 dpi, and analyzed by Western blot and Q-PCR, respectively. Western blot analysis of protein extracts from control infections showed increasing intensity of T-antigen and VP1 (major viral capsid protein) expression from 8 to 15 dpi suggesting a progressive viral infection cycle as expected (Fig 1A and 1B, compare lane 3 and 4). On the other hand, treatment of cells with conditioned medium from uninduced PBMCs showed a partial but significant reduction in T-antigen and VP1 levels at both time points of infection compared to control infections (Fig 1A, compare lane 5 with 3, and lane 6 with 4). However, VP1 levels were increased from 8 to 15 dpi suggesting that conditioned medium from uninduced PBMCs was unable to inhibit progression of the infection cycle (Fig 1A, compare lane 5 with 6). Interestingly, there was a dramatic decrease in both T-antigen and VP1 levels when the cells were treated with conditioned media from induced-PBMCs (Fig 1A and 1B, lanes 7 and 8), and there was no progressive increase in VP1 expression from 8 to 15 dpi suggesting that the viral life cycle was completely blocked. In parallel with Western blot analysis for the expression of VP1, growth medium of the cells were analyzed for viral copy numbers from the same infection in panel A. Consistent with what was observed for T-antigen and VP1 expressions, conditioned medium from induced PBMCs also showed a dramatic reduction in viral copy numbers (Fig 1C). The decrease in viral copy numbers further supports the negative impact of soluble immune mediators on JCV replication. These results suggest a novel signaling between peripheral immune cells and glial cells that inhibits the JCV life cycle.

### Conditioned media from PBMCs inhibits viral replication induced by T-antigen

**Fig 2:**
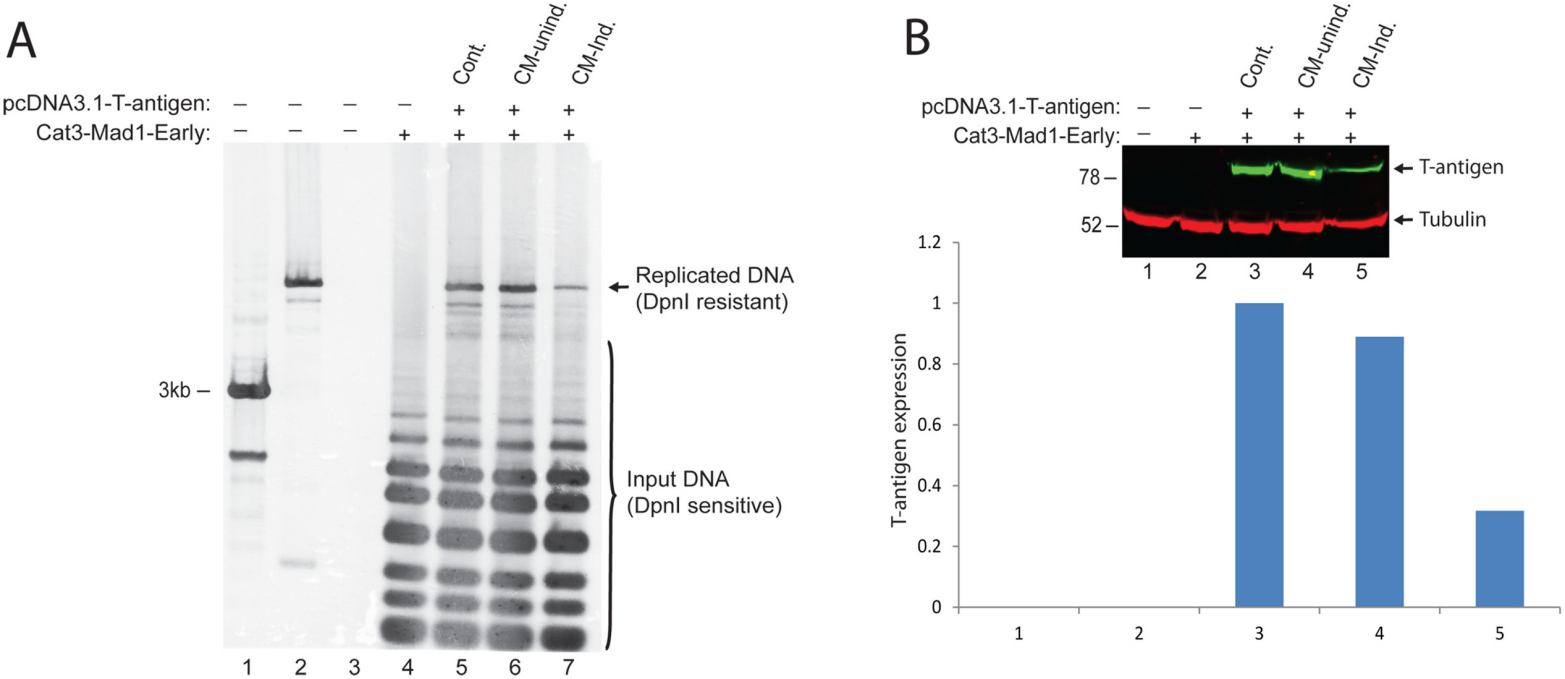
Immune regulators secreted by activated PBMCs inhibit JCV genomic DNA replication mediated by T-antigen. A. JCV genomic DNA replication initiated by Large T-antigen is suppressed by immune mediators secreted by activated PBMCs. PHFA cells were transiently transfected with pBLCAT3-Mad1-Early reporter gene construct contains origin of viral replication and pCDNA3.1- T-antigen expression plasmids. The culture media of the cells were supplemented with conditioned medium obtained from either uninduced or induced PBMCs at a ratio of 50%. Cells were harvested at 72hrs post-transfections, and cell pellets were divided into half. One portion of the cell pellets was utilized for the purification of low molecular weight plasmid DNAs. The DNA samples were first digested with DpnI/BamHI restriction enzymes and separated on 1% agarose gel by electrophoresis. Southern blot analysis of replicated plasmid DNA was performed by DIG-High Prime DNA Labeling and Detection Starter Kit (Roche, US) as described by the Manufacturer. In lane 1, kb-ladder was loaded as molecular weight marker. In lane 2, pBLCAT3-JCV-early plasmid was first linearized with BamHI digestion and loaded as positive control (3ng). Lane 3 was just sample buffer. B. The second half portion of the cell pellets from experiments presented in panel A was used for the preparation of whole cell protein lysates, and western blot analysis were performed for the detection of T-antigen and tubulin expressions. The bar graph represents the normalized expression of T-antigen to tubulin.

In order to elucidate the possible impact of immune mediators on JCV replication, we performed a viral replication assay utilizing pBLCAT3-JCV-early plasmid which contained the entire viral noncoding control region (NCCR) including the origin of viral DNA replication. PHFA cells were transiently transfected with this construct and an expression plasmid encoding viral T-antigen. Cells were harvested for low molecular weight DNA at 4 day post-transfections, and analyzed by DpnI/Southern blot as described in Materials and Methods. As expected, a band corresponding to newly replicated plasmid DNA was only detectable in the presence of T-antigen (Fig 2A, compare lanes 4 and 5). Interestingly, treatment of the cells with conditioned medium from induced but not from uninduced PBMCs showed a significant reduction in the levels of replicated plasmid DNA (compare lanes 6 and 7). These data suggest that immune mediators secreted by activated PBMCs have a negative impact on JCV replication mediated by T-antigen. In parallel to the DNA samples, whole cell protein extracts were also prepared from the same experiments and analyzed by Western blot for the expression of T-antigen (Fig 2B). Surprisingly, conditioned medium from induced PBMCs showed a significant decrease in T-antigen protein levels (compare lane 5 with lanes 3 and 4). Therefore we conclude that the observed suppression of viral DNA replication in Panel A by immune mediators is due to the decrease in T-antigen protein levels rather than a direct suppressive impact on viral genomic replication.

### Post-transcriptional suppression of T-antigen expression by conditioned media from PBMCs

**Fig 3:**
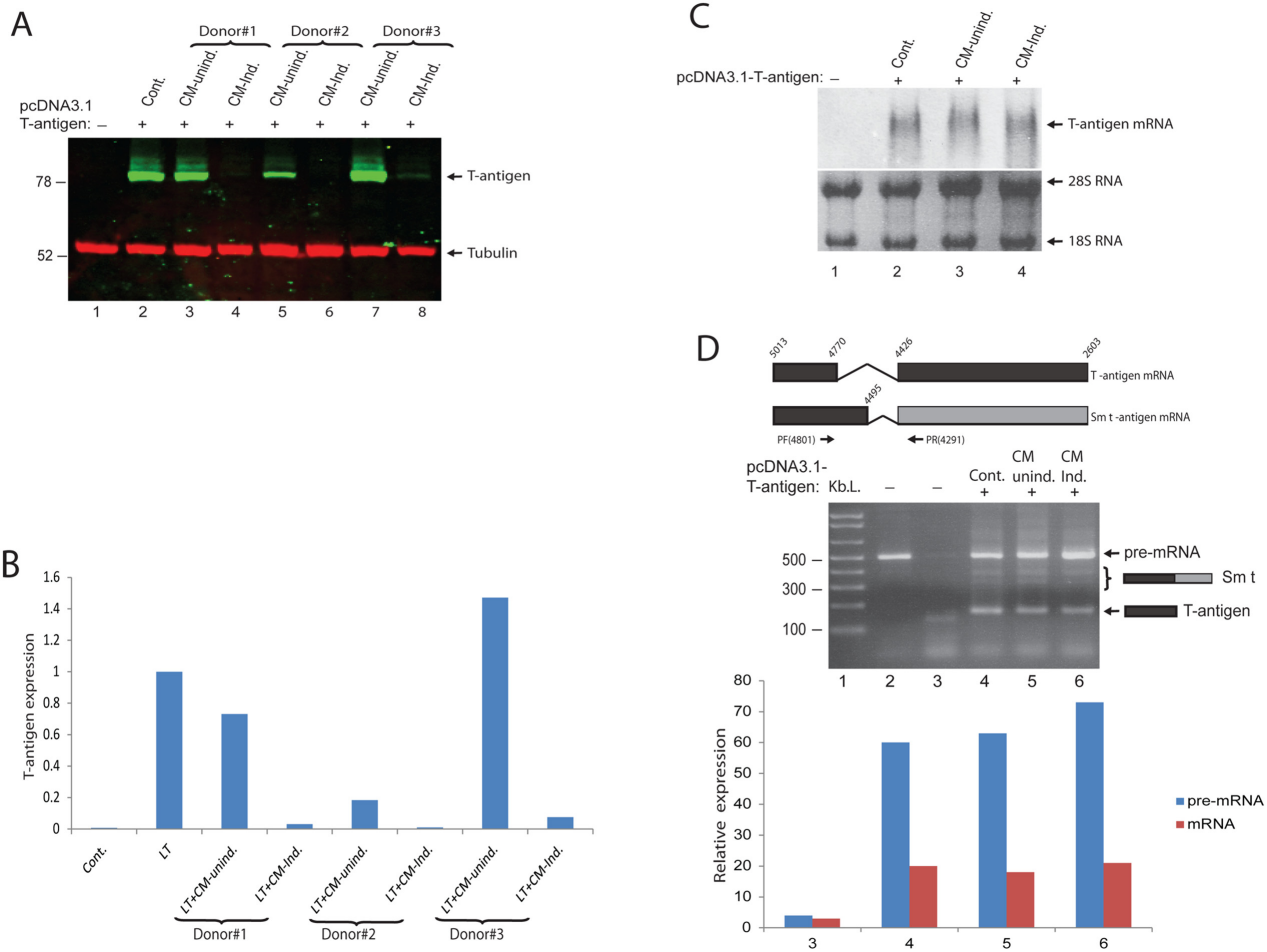
Soluble immune mediators suppress T-antigen protein levels in glial cells. A. T98G cells were transfected with an expression plasmid encoding T-antigen, and directly treated with conditioned media (50% volume) from either uninduced or induced PBMCs obtained from three different donors. Whole cell protein lysates were analyzed by Western blot for T-antigen and tubulin expression. B. Bar graph representation of T-antigen expression normalized to tubulin from panel A. C. Northern blot analysis of JCV-early transcript. T98G cells were transfected with pCDNA3.1- T-antigen expression plasmid which encodes all the possible early genes expressed by alternative splicing including T-antigen and sm t-antigen antigens. The culture media of the cells were supplemented with conditioned media obtained from either uninduced or induced PBMCs at a ratio of 50%. Cells were harvested and for the extraction of total RNA analyzed by Northern blot for the detection of JCV-early mRNA by using DIG Northern Starter Kit as described by the Manufacturer (Roche, US). D. Soluble immune mediators secreted by induced PBMCs do not effect expression of JCV early mRNAs. Total RNA samples from the same experiments presented in Panel C were used for cDNA synthesis by reverse transcriptase reaction. JCV-early region gene products (pre-mRNA, sm t-antigen, and T-antigen) were amplified and separated on a 3% agarose gel and stained with ethidium bromide. Lane 2 was pcDNA3.1-T-antigen plasmid DNA amplified as positive control of unspliced pre-mRA. Lane 3 was untransfected PHFA cell extracts used as a negative control. Schematic structure of JCV early region unspliced and spliced RNAs and, the size of the expected amplification products with a primer set (PF and PR), used for the amplification of JCV gene products are schematized at the top panel. Band intensities of pre-mRNA and mRNA for T-antigen in lanes 3–5 were quantified and presented as bar graph.

To determine the mechanism by which immune mediators secreted by PBMCs dowregulate T-antigen expression, we utilized T98G cells, a human glioblastoma cell line, and a pcDNA 3.1 expression plasmid encoding T-antigen. T98G cells were transfected with pcDNA 3.1-T-antigen construct and treated with conditioned medium from PBMCs prepared from three different donors. Whole cell protein extracts were analyzed by Western blot to determine T-antigen expression levels. As seen in Fig 3A and 3B, conditioned medium from induced PBMCs caused a dramatic decrease in the expression levels of T-antigen, suggesting that T-antigen expression is regulated by neuroimmune signaling. In parallel with the Western blot analysis of T-antigen protein expression, viral early gene transcripts were also analyzed by Northern blotting. As shown in Fig 3C, unlike the protein expression levels, conditioned medium from induced PBMCs did not alter viral early RNA levels, indicating that the observed reduction in T-antigen protein levels is mediated post-transcriptionally. In order to elucidate the possible impact of soluble immune mediators on T-antigen pre-mRNA splicing, RNA samples were processed for RT-PCR by using a primer pair which distinguishes T-antigen mRNA from other early gene alternatively spliced products [25]. As shown in Fig 3D, there was no significant change in pre-mRNA and mRNA levels of T-antigen in cells treated with CM-induced, suggesting that neither transcription nor splicing of early gene products were altered by neuroimmune conditioning.

### Neither autophagy nor proteasomes are involved in T-antigen suppression mediated by neuroimmune signaling

**Fig 4:**
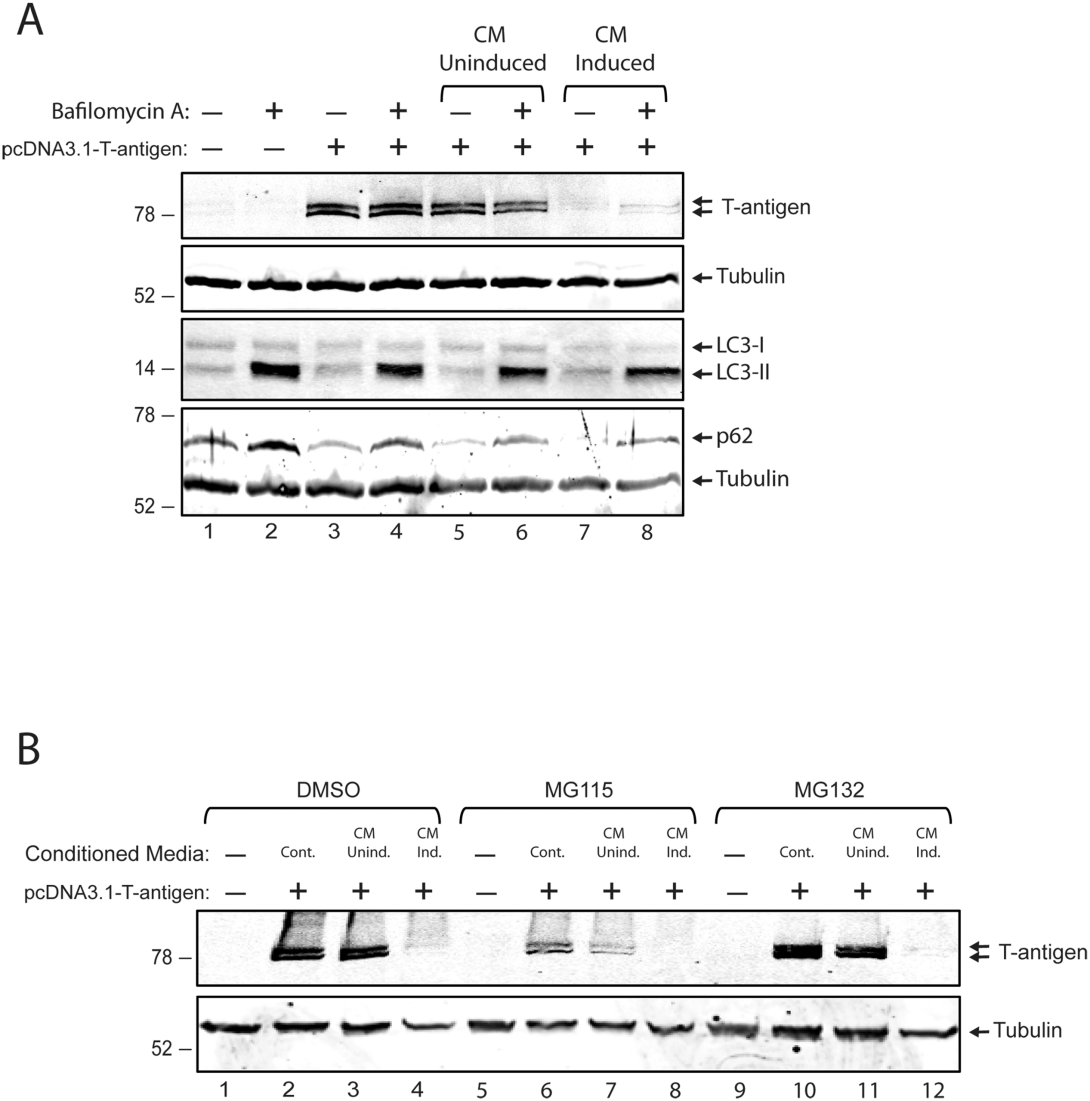
Neither autophagy nor proteasomes are involved in neuroimmune suppression of T-antigen expression. A. T98G cells were transfected with pCDNA 3.1 plasmid encoding T-antigen. Cells were treated with conditioned media obtained from either uninduced or induced PBMCs inn the presence or absence of Bafilomycin A (1uM). Whole cell protein extracts were prepared, and processed for the detection of T-antigen, P62, and LC3 by western blotting. The same membranes were stripped off and probed for tubulin as loading control. B. T98G cells were transfected with pCDNA 3.1 encoding T-antigen. Cells were treated with either CM-induced or uninduced in the absence of DMSO or MG115 (10uM) or MG132 (10uM) as indicated. Whole cell protein lysates were prepared and expression of T-antigen was detected by western blotting. Same membranes were stripped off and probed for tubulin as loading control.

T-antigen is a key viral regulatory protein which acts as a transcription factor to autoregulate its own promoter and drives the downstream steps of the viral life cycle, including DNA replication and expression of the late viral transcripts [26]. The observed suppression of T-antigen expression by soluble immune mediators without altering mRNA levels of the protein suggested a novel mechanism of neuroimmune signaling which controls the expression of T-antigen at the posttranscriptional level. Protein quality control of T-antigen has been shown to be regulated by both proteasomes and autophagy [27], [28]. In order to explore the possible involvement of autophagic signaling in T-antigen suppression mediated by soluble immune mediators, T98G glioblastoma cells were transfected with the pcDNA 3.1-T-antigen expression vector and treated with conditioned medium from either uninduced (CM-uninduced) or induced (CM-induced) PBMCs in the presence of 1uM Bafilomycin A (Sigma Aldrich), a well-known inhibitor of autophagy. As expected CM-induced but not CM-uninduced treatment showed a dramatic suppression in T-antigen protein levels (Fig 4A, lane 7). However, Bafilomycin A treatment of cells did not recover T-antigen suppression mediated by CM-induced (lane 8) suggested that autophagy was not involved in neuroimmune suppression of the protein. Of note, cells treated with Bafilomycin A showed increased LC3-II and p62 levels confirming the effectiveness of autophagic inhibition. On the other hand, possible involvement of proteasomes in neuroimmune suppression of T-antigen expression was also analyzed by treating the cells with MG115 (Sigma Aldrich, 10 uM) or MG132 (Sigma Aldrich, 10 uM), two well-studied inhibitors of the proteosome. Similar to the autophagy results, proteosomal inhibition of cells under the influence of neuroimmune conditioning did not recover T-antigen suppression mediated by soluble immune mediators (Fig 4B) suggesting that the proteasome was also not involved in neuroimmune suppression of T-antigen expression.

### Immune mediators secreted by activated PBMCs inhibit translation initiation in glial cells

**Fig 5:**
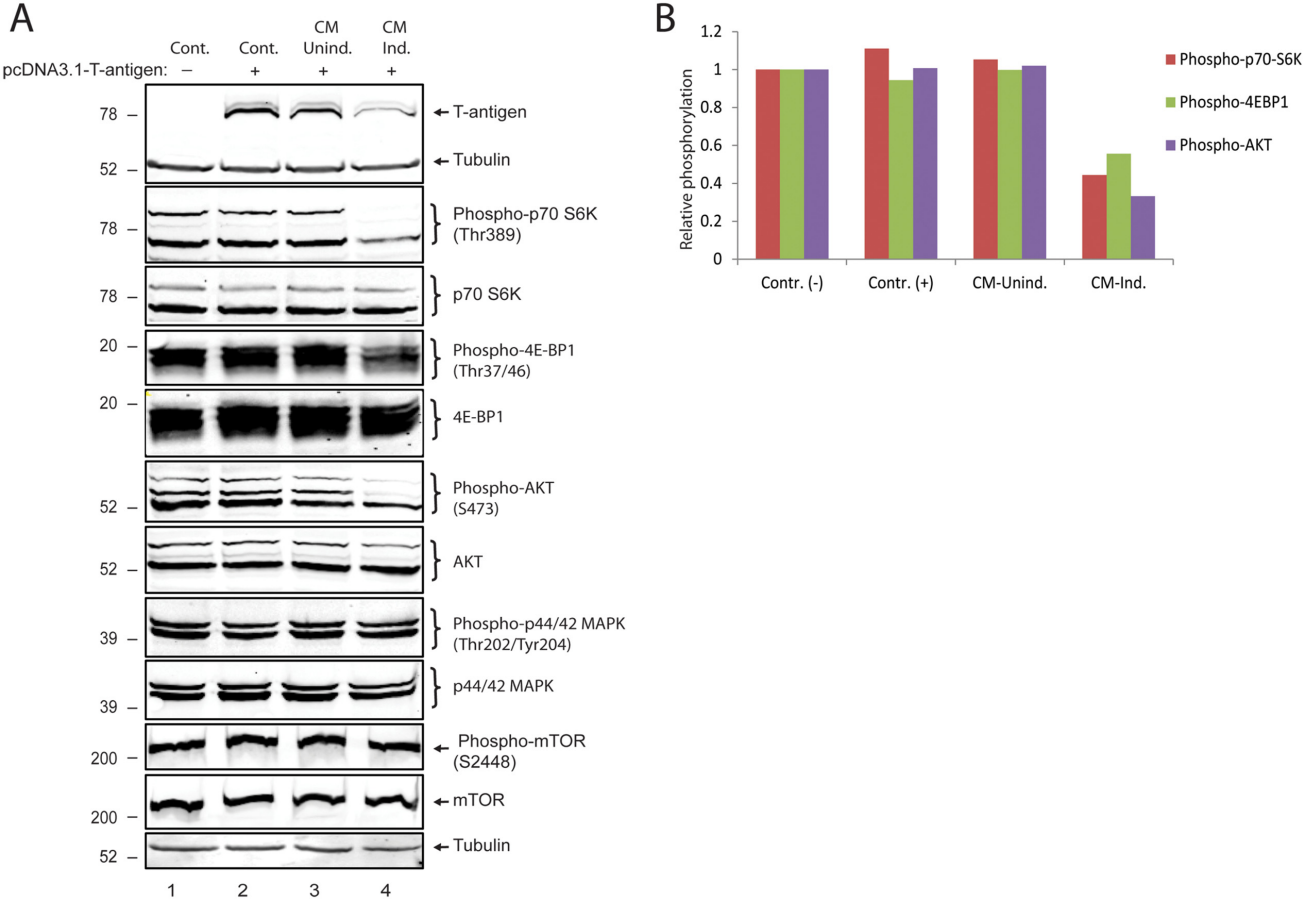
Soluble Immune mediators secreted by activated PBMCs limit translation initiation in glial cells. A. PHFA cells were transfected with an expression plasmid encoding T-antigen and treated with conditioned media from either uninduced or induced PBMCs. Western bot analysis of protein extracts were performed to assess phosphorylated and total protein levels of p70S6K, 4EBP1, AKT, p44/42 MAPK and mTOR. Tubulin was probed as loading control in the same membranes. B. Normalized expressions of phosphor-p70S6K, phosphor-4E-BP1, and phosphor-AKT are shown as bar graph. The phosphorylated protein signals were normalized to total protein levels of each protein from the experiments presented in panel A.

In order to gain more insight into T-antigen suppression mediated by neuroimmune conditioning, possible effects of soluble immune mediators secreted by activated immune cells on protein translational machinery were analyzed by Western blotting. The PI3K/AKT/mTOR pathway controls protein synthesis at the level of translation initiation and ribosome biogenesis. The mammalian target of rapamycin (mTOR) is a serine/threonine protein kinase involved in the regulation of cell growth, cell proliferation, and protein translation. Its involvement in translation consists of its capacity to regulate phosphorylation and inactivation of the repressor of mRNA translation, 4E-BP1 (eukaryotic initiation factor 4E-binding protein), and activation of S6 kinase (S6K) by phosphorylation. We therefore decided to investigate whether this pathway could be affected by soluble immune mediators released from activated PBMCs. As described above, T98G glioblastoma cells were transfected with pcDNA3.1-T-antigen expression plasmid and treated either with CM-uniduced or CM-induced. As expected, CM-induced but not CM-uninduced once again suppressed T-antigen protein levels (Fig 5A). Interestingly, Western blot analysis of S6K phosphorylated at Thr389, 4E-BP1 phosphorylated at Thr37/46, and AKT phosphorylated at S473 showed dramatic decreases without any significant change in their total protein expression levels (Fig 5A and 5B). Surprisingly, neither phosphorylation nor total protein levels of p44/42 MAPK and mTOR were altered under the influence of neuroimmune conditioning. These results have suggested that soluble immune mediators secreted by activated PBMCs inhibit translation initiation in glial cells.

### IFN-γ inhibits T-antigen expression in glial cells

**Fig 6:**
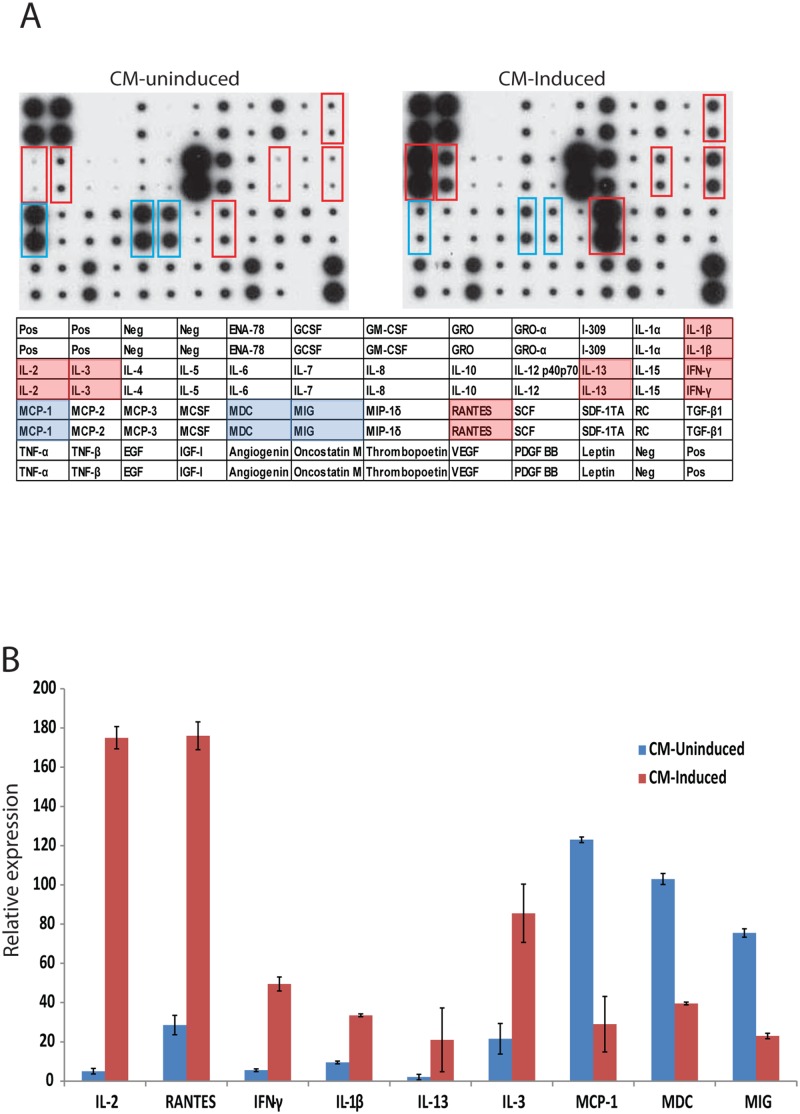
Cytokine array with conditioned media from uninduced and induced PBMCs. **A**. Cytokine profiles of conditioned media from either uninduced (CM-Uninduced) or induced (CM-Induced) PBMCs were compared by cytokine arrays as described by the Manufacturer (RayBiotech, Human Cytokine Array C1, CODE: AAH-CYT-1) Red boxes denote the cytokines with increased levels in CM-induced compared to CM-uninduced. Green boxes denote the cytokines with decreased levels in CM-induced compared to CM-uninduced. B. Quantification of relative expression levels of selected cytokines from the cytokine array studies are represented as bar graph with relative expression.

The observed suppression of T-antigen protein levels by conditioned media from induced but not from uninduced PBMCs has suggested that soluble immune mediators secreted by activated immune cells could possibly be the component of neuroimmune signaling controlling viral gene expression in glial cells. It is expected that induction of PBMCs with PMA/Ionomycin results in increased secretion of proinflammatory cytokines and chemokines into the growth media of the cells which had been used in our study. We therefore analyzed production of multiple cytokines/chemokines utilizing a commercially available antibody-based array (RayBiotech, US) which revealed a robust increase in the expression of IL2, Rantes, IFN-γ, IL-1β, IL-13 and IL-3 levels and a concomitant decrease in the expression of MCP1, MDC and MIG (Fig 6A and 6B).

**Fig 7:**
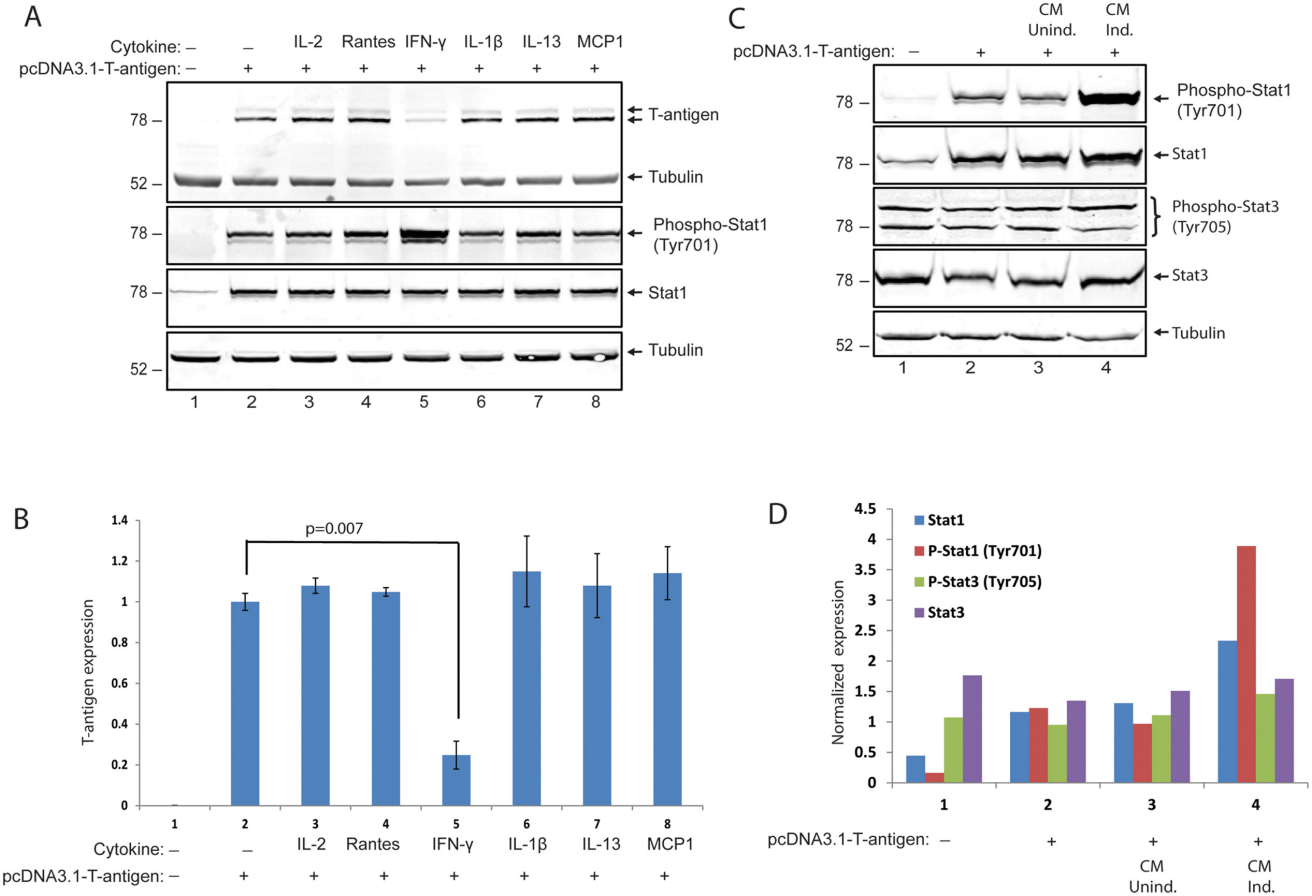
IFN-γ inhibits T-antigen expression in glial cells. A. PHFA cells were transfected with pCDNA 3.1 plasmid encoding T-antigen. Cells were treated with IL2 (6 ng/ml), Rantes (10 ng/ml), IFN-γ (100 ng/ml), IL-1β (5 ng/ml), IL13 (5 ng/ml), and MCP1 (6 ng/ml) for 48hr. Whole cell protein lysates were processed for the detection of T-antigen, phospho-Stat1 and total-Stat1. The same membranes were stripped off and probed for anti-tubulin as loading control. In lane 1, whole cell protein lysates from cells transfected with pcDNA3.1 vector DNA were also processed as controls of transfection. B. Bar graph representation of normalized T-antigen expression signal from panel A. C. Stat1 signaling is induced by soluble immune mediators secreted by activated immune cells. PHFA cells were transfected with an expression plasmid encoding T-antigen and treated with conditioned media from either uninduced or induced PBMCs. Western blot analysis of protein extracts was performed to assess phosphorylated and total protein levels Stat1 and Stat3. Tubulin was probed as loading control in the same membranes. D. Normalized expression levels of phospho-Stat1, total-Stat1, phospho-Stat3, and total-Stat3 from the Western blot studies in panel C were presented as bar graph.

We next investigated the possible effects of selected cytokines/chemokines identified from the array studies on T-antigen expression levels. T98G glioblastoma cells were transfected with a plasmid encoding T-antigen and were treated with IL-2, Rantes, IFN-γ, IL-1β, IL-13 and MCP1 at 12, 24, and 36hrs post-transfection. Whole cell protein extracts were prepared at 48hrs post-transfection, and analyzed by Western blotting. Interestingly, among the all candidate cytokines, only IFN-γ induced a significant suppression of T-antigen protein levels (Fig 7A and 7B). IFN-γ, or type II interferon, is an important cytokine that has critical roles in innate and adaptive immunity [29]. Cellular responses to IFN-γ are activated by its interaction with interferon gamma receptor 1(IFNGR1) and interferon gamma receptor 2 (IFNGR2). The IFNGR intracellular domain contains binding motifs for the Janus tyrosine kinases (Jaks) and the latent cytosolic factors, signal transducers and activators of transcription (Stats). IFN-γ binding induces Jak2 autophosphorylation and activation, which allows Jak1 transphosphorylation by Jak2 [30–32]. The activated Jak1 recruits Stat1 which is phosphorylated near the C terminus at Y701 [33], [34]. To determine whether Jak/Stat1 signaling was induced by IFN-γ treatment in T98G cells, the phosphorylation status of Stat1 at Y701 was analyzed by Western blotting utilizing a phospho-specific antibody. As expected, IFN-γ treatment induced levels of phosphorylated Stat1 compared to control cells with no treatment or to cells treated with other cytokines (IL2, IL-1β, IL13) and chemokines (Rantes and MCP1) (Fig 7A, compare lane 5 with lanes 2–4 and 6–8). Control cells expressing T-antigen resulted in not only higher levels of phosphorylated Stat1 at Y701, but also an increase in total Stat1 protein levels when compared to cells transfected with control plasmid DNA (Fig 7A, compare lanes 2 and 1). To validate the Stat1 activation upon treatment of cells with conditioned medium from induced PBMCs, whole cell lysates from cells transfected with an expression plasmid encoding T-antigen and treated either with CM-induced or CM-un-induced were analyzed by Western blot for total and phosphorylated Stat1 at Y701 and total or phosphorylated Stat3 at Y705. Once again, expression of T-antigen resulted in an increase in total and phosphorylated Stat1 levels. Consistent with IFN-γ treatment, conditioned media from induced but not from uninduced PBMCs caused a further increase in Stat1 phosphorylation at Y701 (Fig 7C and 7D). Neither T-antigen expression nor soluble factors secreted by activated PBMCs caused any significant change in total or phosphorylated levels of Stat3 at Y705 which suggest that Stat1, but not Stat3, is the main downstream component of neuroimmune conditioning in cells expressing T-antigen.

### Neuroimmune suppression of T-antigen expression is mediated by Jak signaling

**Fig 8:**
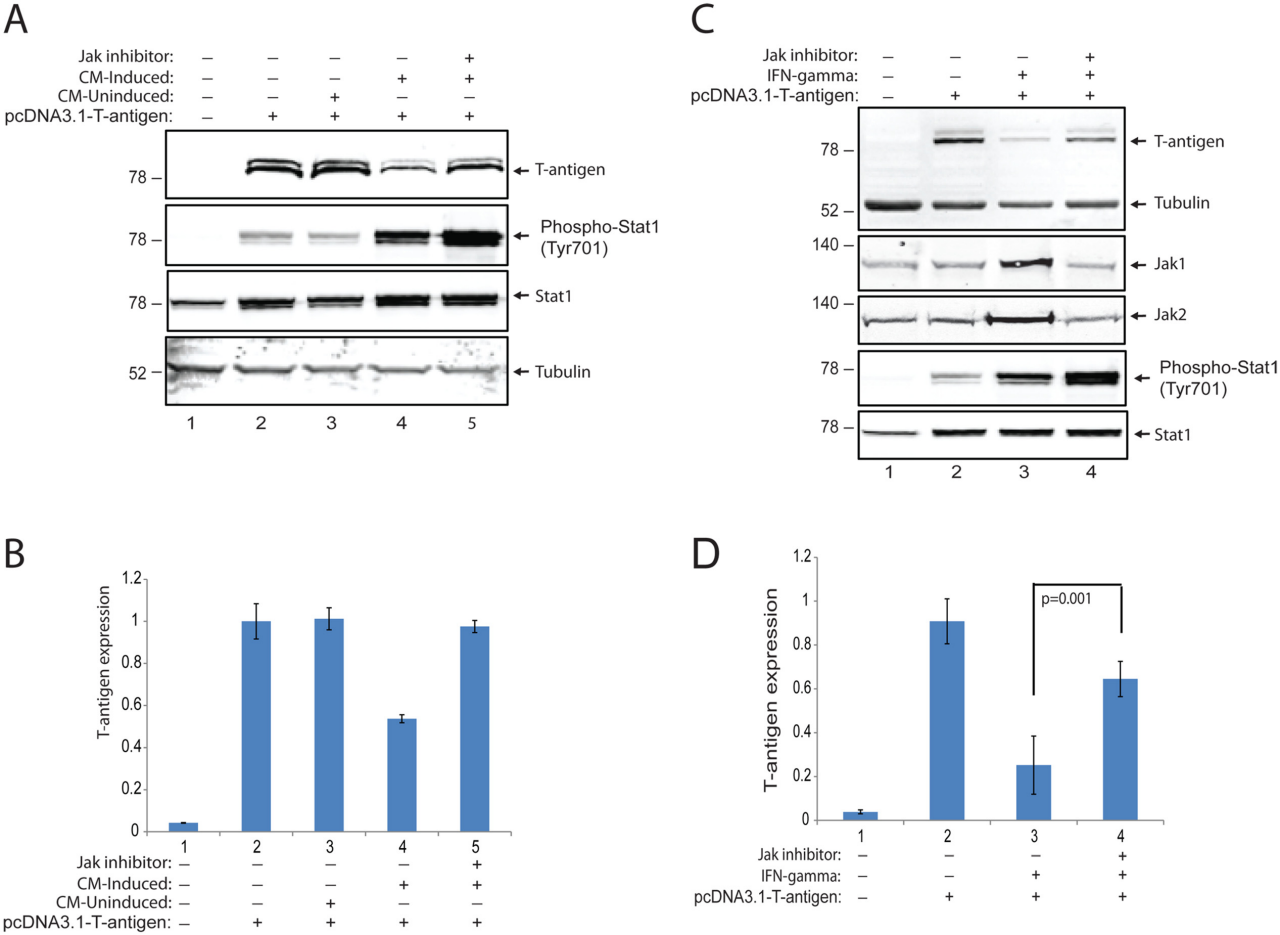
Neuroimmune suppression of T-antigen by IFN-γ is mediated by Jak signaling. **A**. PHFA cells were transfected with pCDNA 3.1 plasmid encoding T-antigen. Cells were treated with either CM-induced or uninduced, and also incubated with a Jak inhibitor (EMD Millipore, #420099, 500 nM). Whole cell protein lysates were processed by Western blot for the detection of T-antigen, phosphorylated-Stat1, and total-Stat1 expressions. B. Normalized T-antigen expression signal from the experiments shown in panel A were represented as bar graph. C. PHFA cells were transfected with pCDNA 3.1 plasmid encoding T-antigen. Cells were treated with IFN-γ and also incubated with the Jak inhibitor. Whole cell protein lysates were processed by Western blot for the detection of T-antigen, Jak1, Jak2, phospho-Stat1, and total-Stat1 expressions. D. Normalized T-antigen expression signal from the experiments shown in panel C were represented as bar graph.

In order to further investigate the role of IFN-γ signaling through interferon gamma receptor (IFNGR) and Janus tyrosine kinases (Jaks) in suppression of T-antigen protein levels, a Jak inhibitor (EMD Millipore #420099, JAK1 (IC50 = 15 nM), JAK2 (IC50 = 1 nM), JAK3 (Ki = 5 nM), was utilized to block the signaling mediated by neuroimmune conditioning. Interestingly, a series of studies revealed that T-antigen expression suppressed by soluble immune mediators was recovered by the Jak inhibitor (Fig 8A and 8B), suggesting that neuroimmune signaling mediated by Jaks was mainly involved in T-antigen suppression in glial cells. Interestingly, treatment of cells with the Jak inhibitor resulted in a further increase in phosphorylation of Stat1 at Y701 in addition to a robust increase caused by conditioned media from induced PBMCs. The observed recovery of T-antigen protein levels by Jak inhibitor with a further increase in Stat1 phosphorylation has suggested that T-antigen suppression may be independent from Stat1 activation. To clarify if recombinant IFN-γ is able to utilize Jak signaling to suppress T-antigen levels, glial cells were transfected with T-antigen expression vector and treated with recombinant IFN-γ in the presence and absence of the Jak inhibitor. As expected, IFN-γ treatment suppressed T-antigen protein levels and induced a robust increase in Jak1, Jak2, and phosphorylated Stat1 at Y701 (Fig 8C). Consistent with conditioned media treatments from induced PBMCs, the Jak inhibitor caused a significant recovery of T-antigen suppression mediated by IFN-γ treatment (Fig 8C and 8D, compare lanes 3 and 4). Moreover, treatment of cells with the Jak inhibitor suppressed the levels of Jak1 and Jak2 induced by IFN-γ and caused a robust increase in Stat1 phosphorylation at Y701 with no alteration of total Stat1 protein levels.

### IFN-γ inhibits JC virus infection in primary human astrocytes

**Fig 9:**
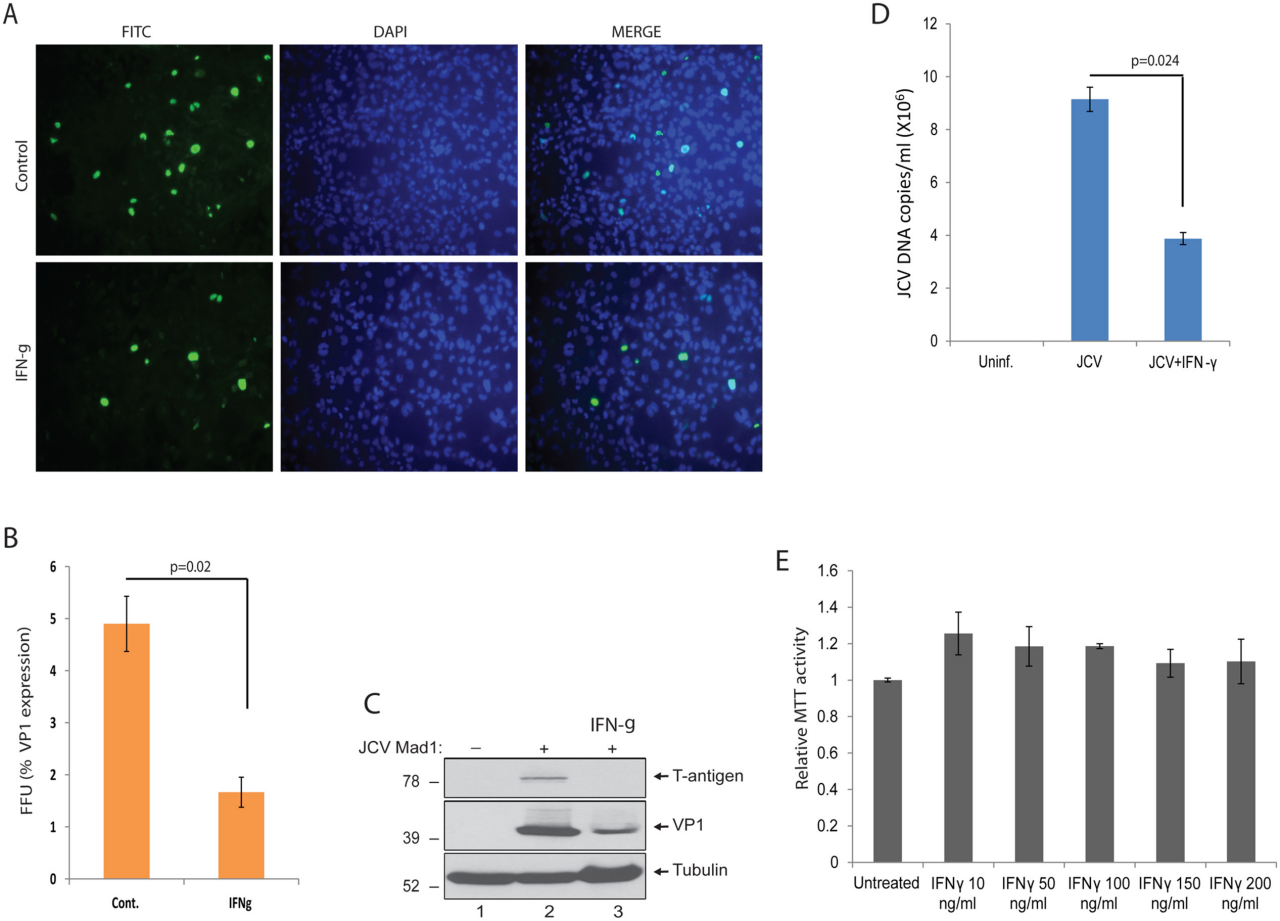
IFN-γ suppresses JCV propagation in PHFA cells. A. PHFA cells were seeded on 2-well chamber slides and infected with JCV Mad1 at a MOI of 1. At 1, 3, and 5 dpi of post-infections, one group of the infected cells was treated with IFN-γ (100 ng/ml). Only 50% of the growth media of the cells were supplemented with fresh media during the course of each IFN-γ treatment. Cells were fixed at 8 dpi with cold acetone/methanol, and processed by immunocytochemistry for the detection of VP1 expression. B. The percentile of VP1 positive cells from panel A were calculated as fluorescence-forming-unit (FFU) and presented as bar graph. C. PHFA cells were infected with JCV Mad1 at a MOI of 1. At 1, 3, and 5 dpi of post-infections, one group of the infected cells was treated with IFN-γ (100 ng/ml). Whole cell protein lysates were prepared at 8 dpi, and analyzed by Western blotting for the detection of T-antigen and VP1. Tubulin was also probed in the same membranes as loading control. D. The growth media of the cells were also collected from the same infections presented in panel A and B, and processed by Q-PCR analysis for the detection of JCV copy numbers. Bar graph represents JCV copy numbers from three independent infection studies. The growth media of uninfected PHFA cells were also processed by Q-PCR and shown in lane 1 with no positivity for JCV. E. Effect of IFN-γ on cellular viability of PHFA cells. PHFA cells were treated with IFN-γ with the indicated dosages for three times at 0, 24 and 48hrs. MTT cell viability assay was performed at 72hrs post-treatments, and relative MTT activities were presented as bar graph.

The observed suppression of T-antigen expression by IFN-γ suggested a possible anti-JCV activity of this cytokine which may be a novel candidate to target the viral lytic infection cycle and treat PML. In order to test potential anti-JCV activity of IFN-γ, we utilized primary human fetal astrocytic (PHFA) cell cultures which are permissive for JCV replication and have been widely utilized by us and others as a cell culture model of JCV infection. PHFA cells were plated in 2-well chamber slides, infected with the Mad1 PML isolate of JCV at an MOI of 1, and treated with IFN-γ (100 ng/ml) at 1, 3, and 5 dpi. Cells were fixed with cold acetone:methanol at 8 dpi, and processed by immunostaining for detection of the viral major capsid protein, VP1. As shown in Fig 9A and 9B, IFN-γ treatment of JCV infected PHFA cells resulted in a significant reduction in the number of the cells expressing VP1. In addition to immunostaining, the impact of IFN-γ treatment on JCV gene expression was also analyzed by Western blotting. As shown in Fig 9C, IFN-γ treatment of PHFA cells infected with JCV suppressed T-antigen expression and resulted in a significant decrease in VP1 levels (Fig 9C). In parallel to VP1 immunostaining and Western blot analysis of viral gene expressions, the growth media harvested from the cells were collected and processed by Q-PCR for the detection of viral copy numbers. Consistent with the decreased levels of the viral gene expression and reduced numbers of the cells infected with JCV, IFN-γ also caused a significant reduction in viral DNA copy numbers as compared to infected controls (Fig 9D). To measure any possible toxicity of IFN-γ treatment, PHFA cells were treated with IFN-γ at different concentrations, and cytotoxicity was determined by MTT viability assay. As shown in Fig 9E, IFN-γ treatment of cells at concentrations ranging from 10 to 200 ng/ml did not have any significant effect on cellular viability. These results have suggested that IFN-γ inhibits JCV infection in glial cells with no toxicity associated, and may be a potential candidate cytokine for the treatment of PML.

## Discussion

Immune surveillance in the CNS is a delicate balance between vigilantly clearing potentially harmful pathogens and minimizing immunological responses which can create damage to normal neurons and glia if left unabated. Although present in small numbers relative to peripheral organs, immune cells trafficking from periphery including T-cells, dendritic cells, and macrophages, continuously inspect the healthy brain for pathogenic agents that would disrupt homeostasis and optimal functioning of this vital organ. Viral infection of the brain, such as enterovirus 71 (EV71) infection of the brain stem, can influence gene expression in peripheral blood mononuclear cells suggesting a possible neuroimmune signaling causing alterations in immune response against CNS pathogens [35]. Therefore interaction between peripheral immune cells and neuroglia becomes highly critical for the suppression and eradication of opportunistic viral infections of the brain.

PML is a neuroimmune disease caused by the neurotropic JCV. Reactivation of JCV in patients with an impaired immune system results in reactivation of the virus from latently infected brain leading to productive infection of oligodendtrocytes and astrocytes by an unknown mechanism [3], [17]. Here, we investigated the possible impact of neuro-immune signaling between peripheral immune cells and brain glia on JCV reactivation and replication of the virus in glial cells. JCV shows a highly restricted species tropism to humans that have made it difficult to develop a suitable animal model for PML. Because there is no established animal model for PML, we utilized human PBMCs and primary human fetal glial cells (PHFG) in cell culture setting as the source of immune mediators and host glial cells in our infection studies. Our experiments have revealed that soluble immune mediators secreted by activated immune cells strongly suppressed JC virus replication. These results suggest a novel signaling mechanism between peripheral immune cells and glial cells that inhibits the JCV lytic infection cycle.

Surprisingly, our results have discovered that soluble immune mediators inhibited viral replication by targeting the major viral regulatory protein, T-antigen, by suppressing its expression levels without altering mRNA levels of the protein. This unexpected suppression of T-antigen was neither associated with autophagic nor proteosomal degradation pathways. Instead, soluble immune mediators had a suppressive effect on translation initiation by causing a decrease in the phosphorylation of p70-S6K, 4EBP1, and AKT. T-antigen is the key viral regulatory protein, which acts as a transcription factor to autoregulate its own promoter and drive the downstream steps of the viral life cycle, including DNA replication and expression of the late viral transcript, which encodes agnoprotein and capsid proteins VP1, VP2, and VP3 [26]. In regulation of viral replication, T-antigen hijacks the host cellular machinery via sequestration of key cellular regulators. T-antigen stabilizes key members of the Wnt signaling pathway, β-catenin and c-myc, resulting in increased nuclear translocation [36–38], which impacts several genes downstream of these transcription factors. T-antigen can also lead to infected cells to evading apoptosis through its interactions with the IGF-1 receptor, which enhances mTOR signaling and expression of the anti-apoptotic protein, survivin [39]. Alternatively, BAG3, another inhibitor of apoptosis, can suppress T-antigen expression and prevent JCV replication [28]. T-antigen has affinity to bind viral origin of replication, and can function like a helicase to unwind viral genome during the replication. Thus, initiation of replication by T-antigen is the essential step in viral replication and a key potential target to block the reactivation of JCV.

The observed suppression of T-antigen protein levels by conditioned media from induced PBMCs has suggested that soluble immune mediators (chemokines and cytokines) secreted by activated immune cells could possibly be the component of neuroimmune signaling controlling viral gene expression. Our array studies using conditioned media from activated PBMCs have revealed several candidate cytokines with possible roles in this regulation. Among them, only IFN-γ showed a robust inhibition of T-antigen expression. IFN-γ, or type II interferon, is an important cytokine that has critical roles in innate and adaptive immunity [29]. Cellular responses to IFN-γ are activated by its interaction with a heterodimeric receptor consist of interferon gamma receptor 1(IFNGR1) and interferon gamma receptor 2 (IFNGR2). The IFNGR intracellular domain contains binding motifs for the Janus tyrosine kinases (Jaks: 1–3 and Tyk2) and the latent cytosolic factors, signal transducers and activators of transcription (Stats: 1–6, including Stat5a and Stat5b). IFN-γ binding induces Jak1 transphosphorylation by Jak2 [30]. The activated Jak1 phosphorylates functionally critical tyrosines on residue 440 of each IFNGR1 chain to form two adjacent docking sites for the SH2 domains of latent Stat1 [31–34]. The receptor-recruited Stat1 pair is phosphorylated near the C terminus at Y701. IFN-γ treatment of glial cells resulted in increased phosphorylation of Stat1 at T701 suggesting involvement of Jak/Stat pathway in the control of T-antigen expression. It has been recently shown that T-antigen of polyomaviruses including JC virus is capable of inducing Stat1 protein levels in mouse embryonic fibroblasts, and its function was attributed to LXCXE motif on the protein [40]. Consistent with these observations, we also reported an increase in both total and phosphorylated Stat1 levels in glial cells expressing JC virus T-antigen. The actual mechanism and functional importance of intrinsic Stat1 activation by T-antigen remains to be determined. To gain more insight into molecular regulation of T-antigen expression by IFN-γ, cells were treated with a Janus Kinase (Jak) inhibitor. Interestingly, Jak inhibitor was able to recover T-antigen expression suppressed by either IFN-γ or neuroimmune conditioning suggested that Jak signaling was the main target of IFN-γ leading to observed suppression of T-antigen expression in glial cells. The unexpected increase in the levels of Stat1 phosphorylation by Jak1 inhibitor in addition to the increase caused by T-antigen expression and IFN-γ treatment suggest that IFN-γ mediated suppression of T-antigen expression may not be associated with increased activation of Stat1.

There is no effective treatment for PML and it is highly fatal within 6–12 months [41]. Currently, the only available option for PML patients is the restoration of underlying immune impairment. The observed suppression of T-antigen expression by IFN-γ has suggested a possible anti-JCV activity of this cytokine which may be a novel candidate to target the viral life cycle. The anti-polyomavirus activity of IFN-γ was previously reported for BK virus (BKV), causative agent of polyomavirus nephropathy in human, and mouse polyomavirus (MPyV) [42], [43]. IFN-γ is capable of inhibiting BKV replication by causing a reduction in the levels of viral gene expression in proximal kidney tubular cells [42]. Similarly, Wilson et al (2011) showed that IFN-γ can reduce expression of MPyV proteins and impair viral replication in 3T3 cells [43]. Consistent with these observations showing anti-BKV and anti-MPyV activity of IFN-γ, our infection studies in glial cells have also suggested a strong anti-JCV activity of this cytokine with no cytotoxicity suggesting its potential for the treatment of PML as well as for the treatment of other polyomavirus-associated disorders.

In summary, our results have revealed a novel neuroimmune signaling mediated by IFN-γ controlling JC virus T-antigen expression in glial cells at the posttranscriptional level, and provide a new avenue of research to understand molecular mechanisms of viral reactivation leading to JCV infection and development of PML in the brain.

## Materials and Methods

### Ethics statement

Human peripheral blood mononuclear cells (PBMC) were isolated from buffy coats (obtained commercially, Biological Specimens, Inc.) in a study approved by the IRB and by the Temple University Institutional Biosafety Committee. Cultures of primary human fetal astrocytes were prepared from human fetal brain tissue obtained under approval of the Temple University Institutional Review Board (IRB). The study was classified as exempt by the IRB, and therefore, per IRB guidelines, a waiver of consent was approved and no informed consent was required or obtained.

### Cell lines and culture

Human derived T98G glioblastoma cell lines cell lines were obtained from American Type Culture Collection (ATCC) and were cultured in Dulbecco's Modified Eagle's Medium (DMEM) supplemented with 10% heat-inactivated fetal bovine serum (FBS) and antibiotics (penicillin/streptomycin, 100 μg/ml). They were maintained at 37°C in a humidified atmosphere with 5% CO2. Primary human fetal astrocytic cells (PHFA) were cultured from fetal brain and provided by the Comprehensive NeuroAIDS Core facility at the Temple University Department of Neuroscience. Human peripheral blood mononuclear cells (PBMC) were isolated from the heparinized blood of buffy coats by density gradient centrifugation on Ficoll-Paque solution (AMERSHAM Biosciences). Briefly, the freshly drawn blood was diluted at a ratio of 1:1 (vol/vol) with PBS at room temperature and 20 ml of the mixture was layered above 20 ml Ficoll-Paque solution (ratio 1:1) in 50 ml tubes. After centrifugation at 1200 g for 30 min at 25°C without break, the mononuclear cell ring with a cloudy appearance were then collected and re-suspended in RPMI media and centrifuged at 2000 rpm for 10 min at room temperature. Pellets were then washed twice with PBS and centrifuged again as described. Finally, PBMCs were washed with RPMI media supplemented with 10% heat-inactivated fetal bovine serum (FBS) and antibiotics (penicillin/streptomycin, 100 μg/ml), and resuspendend in culture media at a concentration of 2 x 10^6^ cells /ml. 48 h after isolation, PBMCs were then divided into two groups. One group was induced with PMA (Sigma-Aldrich, 1.5 ug/ml) and Ionomycin (Sigma Aldrich, 0.1 ug/ml) for 2 hrs, and drug-containing medium was replaced with fresh media. The second group of cells was left uninduced. Both induced and uninduced cells were maintained at 37°C in a humidified atmosphere with 5% CO2. At 48hrs post-induction, growth media of the cells were collected, centrifuged at 3000 rpm for 10 min to remove the debris, and either used fresh for the proposed experiments or stored at −80°C as aliquots for single thawing and usage for future studies.

### Plasmid constructs and viral strains

T-antigen (JCV Mad-1 5013–2603, NC_001699.1), was cloned into an eukaryotic expression vector pcDNA3.1(+) at EcoRI restriction enzyme site and designated as pcDNA3.1–T-antigen previously described [44]. CAT3-Mad1-Early construct which contains JCV NCCR and origin of viral replication was described previously [21]. pBlue-Mad1 WT plasmid was described previously [21], [25]. Briefly, Mad1 strain of JCV was linearized by BamH1 digestion from pJCV, and cloned into the pBlueScript KS (+) vector.

### JCV virus infection of PHFA cultures

JC virus infection of PHFA cultures was performed as previously described [21], [25], [28]. Briefly, PHFA cells were cultured and plated at a confluence of 1 x 10^6^ cells per T75-cm^2^ tissue culture flask, and transfected/infected with neurotropic Mad-1 strain of JC virus DNA (10μg/flask) using Fugene6 transfection reagent as indicated by the manufacturer (Roche). The growth media of the cells were supplemented with conditioned media from PBMC cultures at 3, 6, 9, and 12 days post-infections (dpi) at a concentration of 1:1 (1 volume PHFA media was mixed with 1 volume conditioned media from PBMCs). At 8 and 15 dpi, the growth media of the cells were collected and analyzed by Q-PCR for viral copy numbers. The cells were trypsinized and processed for whole cell protein extraction for Western blot analysis of the major viral capsid protein VP1 (pAb597). For the IFN-γ treatment studies, PHFA cells were cultured in 2-well chamber slides and infected with the Mad-1 strain of JC virus at a MOI of 1 and were treated with IFN-γ (100 ng/ml) at 1, 3, and 5 dpi. Cells were fixed with cold acetone:methanol at 8 dpi, and processed by immunostaining for the detection of major capsid protein, VP1. The growth media of the cells were also collected at 8 dpi and processed by Q-PCR analysis for the detection of viral copy numbers.

### Quantitative-PCR (Q-PCR) analysis of JCV copy numbers in growth media

Culture media containing the viral particles was collected and centrifuged for 10 minutes at 13,000 rpm in order to remove cellular debris. Supernatants were incubated at 95°C for 10 minutes for inactivation of the virus. Ten microliters of the medium was used as a template in the Q-PCR reactions. The standard curve was obtained after serial dilution of pJCV, a plasmid containing the whole genome of the JCV Mad-1 strain. All Q-PCR analyses were done by using Lightcycler 480 (Roche). Primers were JCV Q-PCR-forward: 5′-AGTTGATGGGCAGCCTATGTA-3′ and JCV Q-PCR-reverse: 5′- TCATGTCTGGGTCCCCTGGA-3′. The probe for the Q-PCR was 5′-/5HEX/CATGGA TGCTCAAGTAGAGGAGGTTAGAGTTT/3BHQ_1/-3′. Each reaction was run with both positive and negative controls and each sample was tested in triplicate.

### Non-radioactive Southern blotting and DpnI assay

PHFA cells were plated in 60 mm tissue culture flasks and transiently transfected with pcDNA3.1–T-antigen expression plasmid and pBLCAT3-JCV-early plasmid which contained whole viral noncoding control region (NCCR) including origin of viral replication. Cells were harvested for the isolation of low molecular weight DNA (QiagenMiniprep Kit) at 4 day post-transfections. Low molecular weight DNA purified from JCV-infected cells were digested with DpnI and BamHI enzymes, separated on 1% agarose gel and then transferred to a nylon membrane. Replicated viral DNA was visualized using a DIG-High Prime DNA Labeling and detection Kit (Roche, US) according to the Manufacturer’s instructions. The whole pBLCAT3-JCV-early plasmid was linearized by BamHI digestion, labeled with DIG, and used as probe for the hybridization of the membranes.

### Non-radioactive Northern blotting

T98G cells were plated in 60 mm tissue culture flasks, transfected with pcDNA3.1–T-antigen expression plasmid, and treated with conditioned media from PBMCs. Total RNA was isolated from cells (Qiagen RNeasy mini Kit), fractionated on a 1% agarose gel and transferred onto a nitrocellulose membrane. T-antigen transcript was visualized using a DIG Northern Starter Kit (Roche, US) according to the Manufacturer’s instructions. A PCR fragment corresponding to coding region of T-antigen (JCV Mad-1 5013–2603, NC_001699.1) was used as probe to detect T-antigen message.

### Western blot analysis

Whole-cell pellets from PHFA or T98G cells were washed with PBS and lysed with TNN lysis buffer (40 mM Tris-HCL pH 7.4, 150 mM NaCl, 1 mM DTT, 1 mM EDTA, 1% NP40, and 1% protease inhibitors cocktail). Protein extracts were eluted with Laemmli sample buffer, heated at 95°C for 10 min, resolved by SDS—PAGE and transferred to reinforced supported nitrocellulose membranes (Whatman, Germany) for 2 h at 4°C in a transfer buffer containing 25 mM Tris (pH 7.4), 200 mM glycine, and 20% methanol. Membranes were blocked for 1 h at room temperature with 10% nonfat dry milk in 1 × phosphate-buffered saline (PBS) with 0.1% Tween-20 (PBST), washed and incubated with primary antibodies for 2–4 h in 5% nonfat dry milk. The blots were subsequently washed three times and incubated with IRDye 800CW goat anti-mouse and IRDye 680RD goat anti-rabbit secondary antibodies and visualized with an Odyssey CLx Imaging System (LI-COR, Inc.,). The following antibodies were used for Western blot: α-VP1 Mouse monoclonal antibody (pAb597) against JCV capsid protein VP1; Anti-SV40-T Antigen (pAb-416, Calbiochem); anti-β-Tubulin (LI-COR); anti-LC3 (Santa Sruz Biotechnology), anti-P62 SQSTM1 Antibody (H-290, Santa Cruz Biotechnology). The following antibodies were purchased from Cell Signaling Technology; anti-phospho- p70S6k1 (Thr389); anti-p70S6k1, anti-phospho-4E-BP1 (Thr37/46); anti-4E-BP1, anti-phospho-AKT (Ser473), anti-AKT, anti-phospho- p44/42 MAPK (Thr202/Tyr204), anti-p44/42 MAPK(Erk1/2), anti-phospho-mTOR (Ser2448); anti-mTOR (7C10), anti-phospho-STAT1 (Tyr701); anti-STAT1; anti-phospho-STAT3 (Ser727), anti-STAT3 (79D7), anti-Jak1, and anti-Jak2.

### Cytokine arrays

PBMCs were isolated from buffy coat preps and either induced or left un-induced as described above. Both induced and un-induced cells were maintained at 37°C in a humidified atmosphere with 5% CO2. At 48hrs post-induction, growth media from induced or un-induced cells were collected, centrifuged at 3000 rpm for 10 min to remove the cellular debris. Cytokine/chemokine profiles of conditioned media were determined by using a commercially available kit (RayBiotech INc., human cytokine array C3, code: AAH-CYT-3) according to the manufacturer's instructions.

### RT-PCR

Total cellular RNA was extracted from PHFA cells transfected with pCDNA 3.1-T-antigen expression plasmid by using Qiagen RNeasy kit according to the manufacturer's recommendations. After treatment with DNase I, followed by phenol/chloroform extraction and ethanol precipitation, cDNAs were synthesized using M-MuLV reverse transcriptase. RNA templates were removed by RNase H digestion. A total of 100 ng cDNA was used as template for PCR reactions. Viral transcripts from JCV infected cells were determined by using following primers in RT-PCR reactions. PF (Mad-1 4801): 5′- CCTGATTTTGGTACATGGAA -3′ and PR (Mad-1 4291): 5′-GTGGGGTAGAGTGTTGGGATCCT -3′. Amplified gene products were resolved on a 3% DNA-agarose gel.

### Immunocytochemistry

PHFA cells were seeded in two-well chamber slides and infected with Mad-1 strain of JC virus at a MOI of 1. At 8 dpi, cells were fixed with cold acetone/methanol (1/1) for 2 minutes and washed three times with 1XPBS. Cells were treated with a 5% BSA solution, followed by incubation with anti-VP1 monoclonal antibody. Cells were then incubated with FITC-conjugated secondary antibody, mounted with aqueous mounting medium with DAPI, and examined under immunoflourescence microscope.
